# Supplementary material for: A Translational Approach to Spinal Neurofibromatosis: Clinical and Molecular Insights from a Wide Italian Cohort
Source: Cancers (Basel). 2022 Dec 22;15(1):59. doi: 10.3390/cancers15010059 (PMC9817775; doi:10.3390/cancers15010059)
Supplement: Supplementary file 1 [file cancers-15-00059-s001.zip › Figure S1.pdf]

Family 1

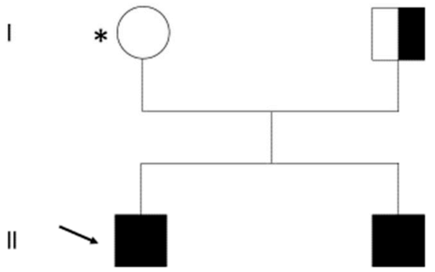

Family 2

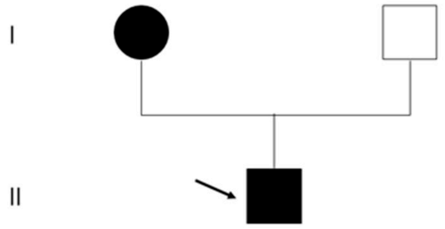

Family 3

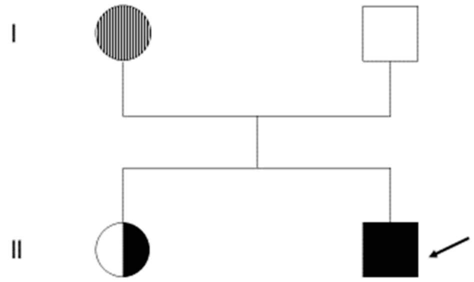

Family 4

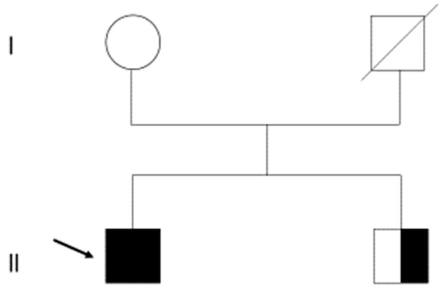

Family 5

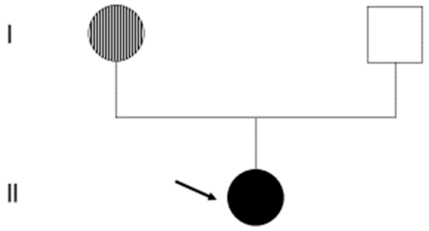

Family 6

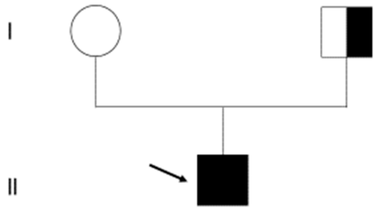

Family 7

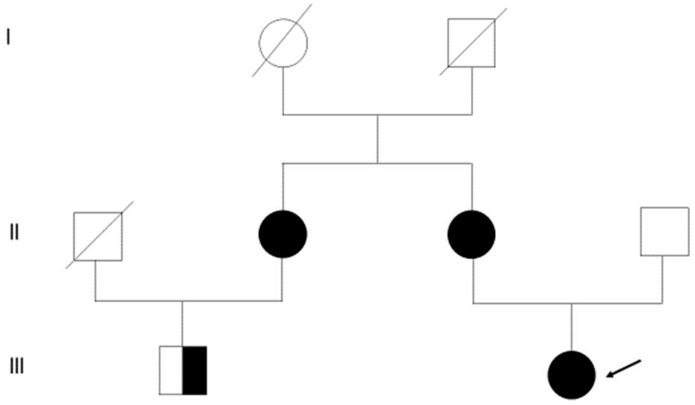

Family 8

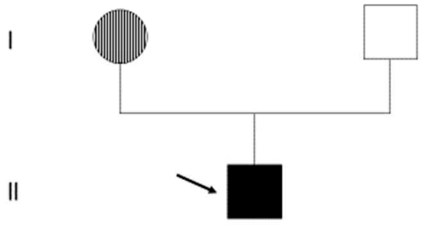

Family 9

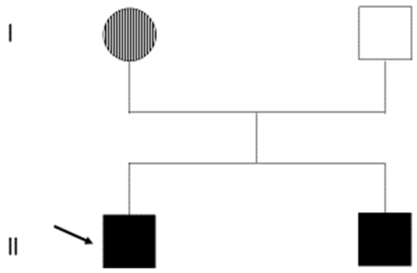

Family 10

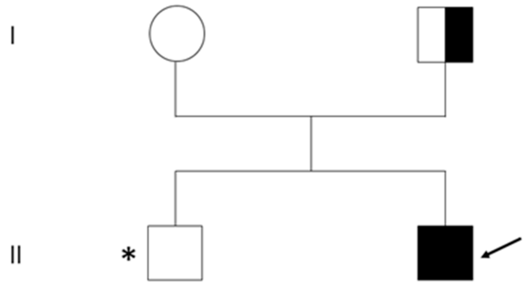

Family 11

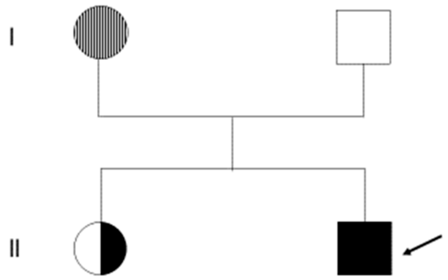

Family 12

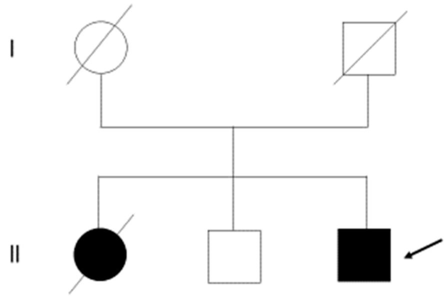

Family 13

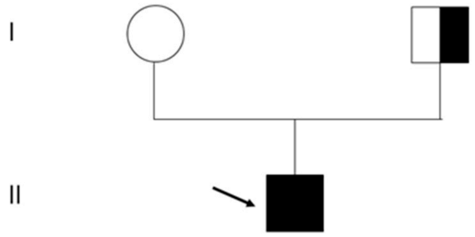

Family 14

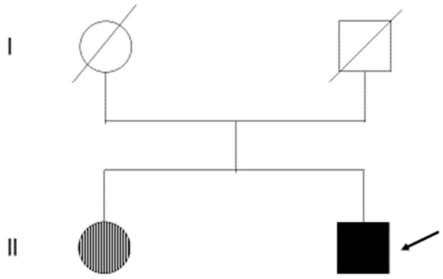

Family 15

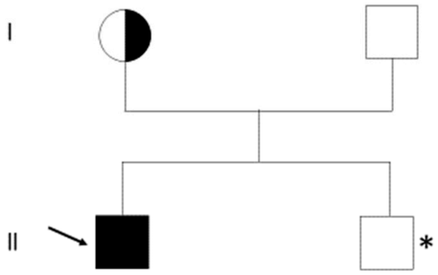

Family 16

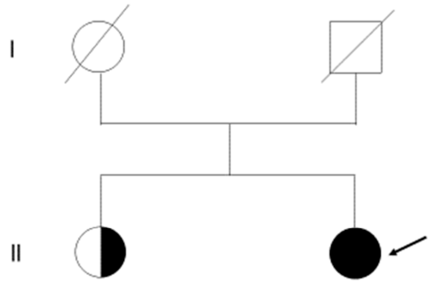

Family 17

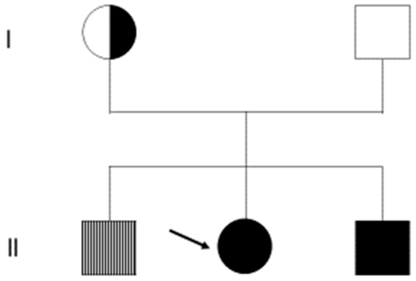

Family 18

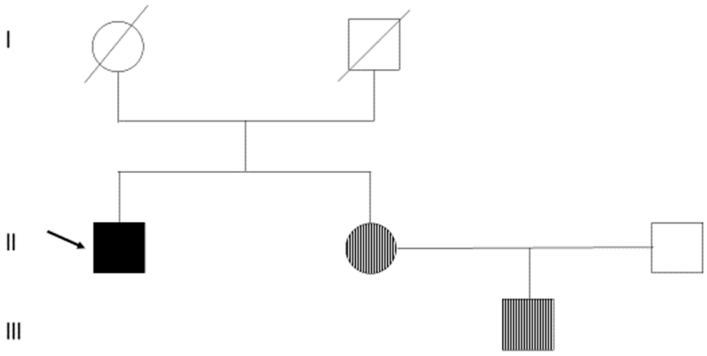

Family 19

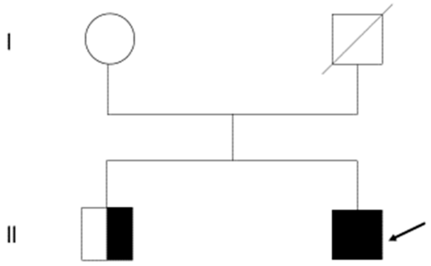

Genetic pedigrees of the 19 SNF families. Here are shown the relationships among the members of each family and their phenotypes. In each family is present at least one member with SNF and the probands are indicated with the arrow. Squares represent males and circles represent females. Empty shapes represent healthy members, while filled shapes represent members affected by NF1:

- Black-filled shapes represent the complete spinal phenotype (SNF), inclouding all the probands
- Half black-filled shapes represent the "partial spinal" phenotype (MNFSR)
- Patterned shapes represent the classical phenothpe
- Barred shapes represent dead patients or members of the family whom phenotype is unknown

* represents uncertain diagnosis, for example patients just with CALs, who do not satisfy the diagnostic criteria for NF1

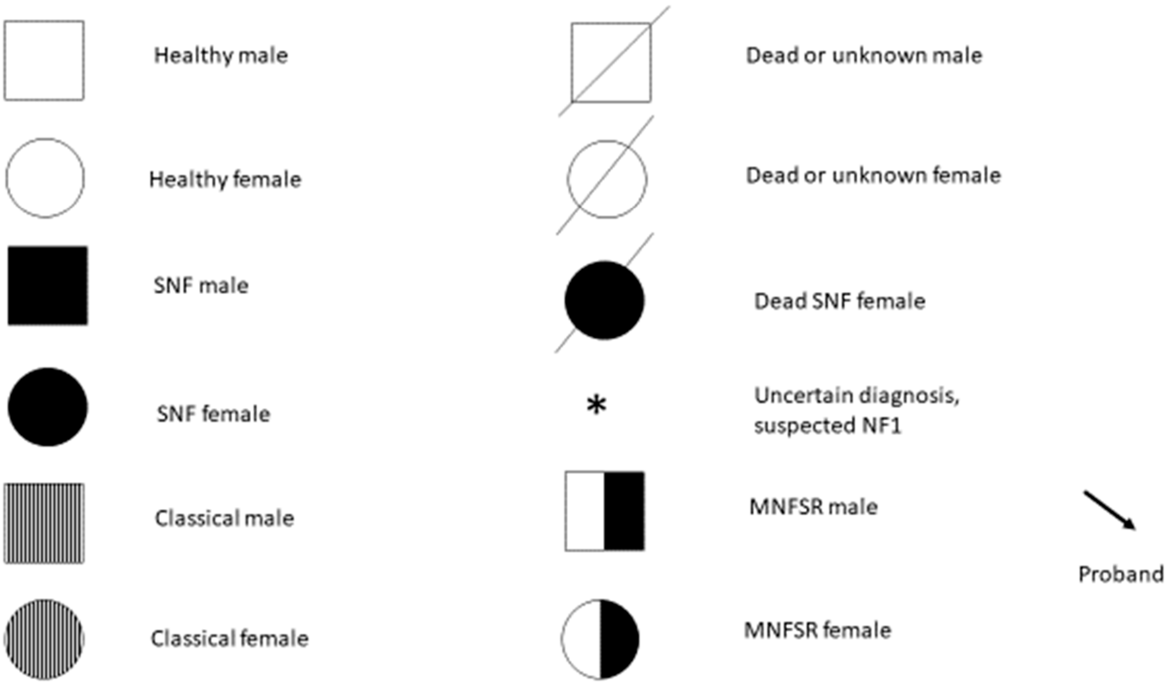

Figure S1. SNF family pedigrees.
