# Supplementary material for: A Translational Approach to Spinal Neurofibromatosis: Clinical and Molecular Insights from a Wide Italian Cohort
Source: Cancers (Basel). 2022 Dec 22;15(1):59. doi: 10.3390/cancers15010059 (PMC9817775; doi:10.3390/cancers15010059)
Supplement: Supplementary file 1 [file cancers-15-00059-s001.zip › File S1.pdf]

NGS analysis

In order to identify the pathogenic *NF1* variants of the patients' populations, we used two different custom targeted resequenching panels (NGStr2, Table A1; NGStr3, Table A2), produced by Agilent Technologies (SureSelect XT panel). The NGStr2 and NGStr3 panels include the coding regions (10 bases from the 3 'end and 10 bases from the 5' end) and the 5 'UTR and 3' UTR regions of 285 genes, of which 19 associated with RASopathies (including *NF1*), 64 belonging to the Ras pathway, 120 coding for neurofibromin interactors, 73 present in the deletion range 17q11.2 and 9 associated with tumours. Before capturing the target region, the DNA libraries were prepared by Agilent Technologies SureSelectXT Target Enrichment System for Illumina Paired-End Multiplexed Sequencing Library and the selection protocol was applied to 3 µg of gDNA for each sample. DNA libraries were hybridized to the Capture Library (<3Mb) containing the panel probes and incubating in a thermal cycler for 24 hours at 65 ° C.

Libraries with DNA concentration > 3 ng/µl were pooled. The final volume of each pool was then corrected to the concentration of 4 nM and the sequencing was performed using MySeq (NGStr2, 2x300 bp) and NextSeq 550 (NGStr3, 2x150 bp) sequencers (Illumina, San Diego, CA, USA).

Read quality assessment and trimming for length at 200 bp (NGStr2) were obtained by means of FastQC (v. 0.11.8; http://www.bioinformatics. babraham.ac.uk/projects/fastqc/) and Trimmomatic (v. 0.36; http://www.usadellab.org/cms/index.php?page=trimmomatic), respectively. Then, the QC-checked paired end (PE) reads of each sample were mapped to NCBI human reference genome (build GRCh37) using BWA-MEM aligner (0.7.10-r789, https://bio-bwa.sourceforge.net/). The mapping was done allowing for maximum 3 mismatches and with other default parameters of BWA. Using Samtools (http://www.htslib.org/), we then re-move the duplicate reads due to PCR amplification during library preparation. For each sample, we retain only high quality (HQ) alignments in sorted BAM files (HQ-BAM) by filtering out unmapped reads and those alignments with mapping quality (MAPQ) less than 15. These high-quality alignments (HQ-BAMs) are then checked for overall mapping statistics (mapping-QC) by an in-house script.

After that, GATK software (v. 3.4, https://gatk.broadinstitute.org/) was used to perform quality score recalibration (using the TableRecalibration walker), local realignment around known indels (using the In-delRealigner walker) and variant calling (by the HaplotypeCaller walker) for both single nucleotide variants (SNVs) and insertions/deletions (indels). Poorly confident variants having QUAL < 150, Fisher Strand (FS) strand bias > 60 for SNV and > 200 for indels, or three SNVs within 10 base-windows were flagged for removal in the FILTER field of the VCF file.

Table A1. List of NGStr2 panel genes

APP	*ADAP2*	*GOSR1*	*TMEM199*	A2ML1	RAC1
CALM1	*ATAD5*	*IFT20*	*TMEM97*	BRAF	RAF1
CASK	*COPRS*	*KIAA0100*	*TMIGD1*	CBL	RASA1
CDC5L	*CRLF3*	*MYO18A*	*TNFAIP1*	GAB1	RASA2
DYNC1H1	*EVI2A*	*NEK8*	*TP53I13*	GAB2	RASA3
GRIN1	*EVI2B*	*NSRP1*	*TRAF4*	GRB2	RASA4
GRIN2B	*LRRC37B*	*NUFIP2*	*UNC119*	HRAS	RASAL1
MAPK3	*NF1*	*PHF12*	*VNT*	KRAS	RASAL2
PML	*OMG*	*PIGS*	*ASIC2*	LIMK2	RASAL3
SDC1	*RAB11FIP4*	*PIPOX*	*C17orf75*	LRP1	RASGRP1
SDC2	*RNF135*	*POLDIP2*	*CDK5R1*	LZTR1	RASGRP2
SDC3	*SUZ12*	*PROCA1*	*MYO1D*	MAP2K1	RASGRP3
SDC4	*TEFM*	*RAB34*	*PSMD11*	MAP2K2	RASGRP4
SUMO1	*ABHD15*	*RPL23A*	*RHBDL3*	MAP3K1	RIT1
YWHAB	*ALDOC*	*SARM1*	*RHOT1*	MAPK1	RRAS
YWHAZ	*ANKRD13B*	*SDF2*	*SPACA3*	MRAS	RRAS2
	BLMH	*SEZ6*	*TMEM98*	NRAS	SHC1
	CORO6	*SGK494*	*ZNF207*	PAK1	SHC2
	CPD	*SLC13A2*		PAK2	SHC3
	CRYBA1	*SLC46A1*		PAK3	SHC4
	DHRS13	*SLC6A4*		PAK4	SHOC2
	EFCAB5	*SPAG5*		PAK6	SOS1
	ERAL1	*SSH2*		PAK7	SOS2
	FAM222B	*SUPT6H*		PTPN11	SPRED1
	FLOT2	*TAOK1*			SYNGAP
	FOXN1	*TBC1D29*			
	GIT1	*TIAF1*			
		TLCD1			

In green, the neurofibromin interactors; in blu, the genes belonging to the 17q11 microdeletion interval, in red the genes belonging to the Ras pathway

Table A2. List of NGStr3 panel genes

A2ML1	CLK1	NOSIP	TNFSF13B	KIAA0100	TNFAIP1	RALGDS
BRAF	DCLK1	NSFL1C	TOP1	LRRC37B	TP53I13	RAP1A
CBL	DENND1A	NXF1	TOP2A	MYO18A	TRAF4	RAPGEF2
GRB2	DENND4A	OSBPL6	TOP3A	MYO1D	UNC119	RAPGEF5
LRP1	DNAJC7	P4HA3	TRAF6	NEK8	UTP6	RASA1
LZTR1	DYNC1H1	PDE4DIP	USP21	NSRP1	VTN	RASA2
MAP2K1	EFNB2	PHLDB2	VCP	NUFIP2	ZNF207	RASA3
MAP2K2	EIF4E2	PLEKHA7	VSIG1	OMG	ABL1	RASA4
MAPK1	EPHA1	PML	VSIG4	PHF12	AKT1	RASAL1
PTPN11	EPHA2	POLR2A	YWHAB	PIGS	ARF6	RASAL2
RAC1	ESR2	POU2F1	YWHAE	PIPOX	BAD	RASAL3
RAF1	FAF2	PRKACA	YWHAH	POLDIP2	BCL2L1	RASGRP1
RIT1	FAM110B	PTEN	YWHAZ	PROCA1	CDC42	RASGRP2
SHOC2	FAM174A	PTPN13	ZBTB21	PSMD11	CHUK	RASGRP3
SOS1	FAM53C	PTPN14	ZNF638	RAB11FIP4	ELK1	RASGRP4
SOS2	GIGYF1	RALGPS2	ZUFSP	RAB34	ETS1	RELA
SPRED1	GIGYF2	RTKN	ABHD15	RHBDL3	FASL	RGL1
SPRY1	GRB7	SCN3B	ADAP2	RHOT1	FOXO4	RGL2
NF1	GRIN1	SDC2	ALDOC	RNF135	GAB1	RHOA
ACTB	GRIN2B	SDC3	ANKRD13B	RPL23A	GAB2	RIN1
ADCY8	HCN1	SDC4	ASIC2	SGK494	HRAS	RIN2
MLLT4	HDAC4	SH3PXD2A	ATAD5	SARM1	KRAS	RIN3
AGAP2	HLA-DPA1	SH3RF3	BLMH	SDF2	LIMK2	RRAS
ANKRD34A	HTR6	SHANK3	C17orf75	SEZ6	MAP3K1	RRAS2
APP	INPP5E	SIGLECL1	CDK5R1	SLC13A2	MRAS	SDC1
ATF2	KCTD3	SIPA1L1	COPRS	SLC46A1	NFKB1	SHC1
BRCA1	KIF13B	SIRT7	CORO6	SLC6A4	NRAS	SHC2
CA14	KIF1C	SLAMF1	CPD	SPACA3	PAK1	SHC3
CALM1	KSR1	SMARCA4	CRLF3	SPAG5	PAK2	SHC4
CAMSAP2	LIMA1	SMARCD1	CRYBA1	SSH2	PAK3	SYNGAP1
CASK	LPIN3	SOX4	DHRS13	SUPT6H	PAK4	CDKN2A
CAV1	LRFN1	SRGAP2	EFCAB5	SUZ12	PAK6	EGFR
CBY1	MAGI1	SRSF12	ERAL1	TAOK1	PAK7	IKBKG
CCDC8	MAP2K3	STARD13	EVI2A	TBC1D29	PIK3CA	PLA1A
CD274	MLK4	SUMO1	EVI2B	TEFM	PIK3CB	RAPH1
CD79B	MAPK3	SYDE1	FAM222B	TIAF1	PIK3CD	TIAM1
CDC25B	MAPKAP1	TANC2	FLOT2	TLCD1	PIK3R1	TRAP1
CDC25C	MAST3	TBPL1	FOXN1	TMEM199	PLD1	VDR
CDC5L	MYC	TESK2	GIT1	TMEM97	RALA	ANRIL
CDK16	NADK	TGOLN2	GOSR1	TMEM98	RALB	DDAH1
CGN	NAV1	TIRAP	IFT20	TMIGD1	RALBP1	

In black, the genes already associated with RASopathies; in green, the neurofibromin interactors; in blu, the genes belonging to the 17q11 microdeletion interval; in red; the genes belonging to the Ras pathway; in grey, the genes associated with tumours
